# Machine learning nonresponse adjustment of patient-reported opioid consumption data to enable consumption-informed postoperative opioid prescribing guidelines

**DOI:** 10.1016/j.sipas.2022.100098

**Published:** 2022-06-10

**Authors:** Chris J. Kennedy, Jayson S. Marwaha, Brendin R. Beaulieu-Jones, P. Nina Scalise, Kortney A. Robinson, Brandon Booth, Aaron Fleishman, Larry A. Nathanson, Gabriel A. Brat

**Affiliations:** a Department of Surgery, Beth Israel Deaconess Medical Center, 110 Francis Street, Suite 2G, Boston, MA 02215, USA; b Center for Precision Psychiatry, Massachusetts General Hospital, Boston, MA, USA; c Department of Biomedical Informatics, Harvard Medical School, Boston, MA, USA; d Department of Emergency Medicine, Beth Israel Deaconess Medical Center, Boston, MA, USA

## Abstract

**Background:**

Post-discharge opioid consumption is a crucial patient-reported outcome informing opioid prescribing guidelines, but its collection is resource-intensive and vulnerable to inaccuracy due to nonresponse bias.

**Methods:**

We developed a post-discharge text message-to-web survey system for efficient collection of patient-reported pain outcomes. We prospectively recruited surgical patients at Beth Israel Deaconess Medical Center in Boston, Massachusetts from March 2019 through October 2020, sending an SMS link to a secure web survey to quantify opioids consumed after discharge from hospitalization. Patient factors extracted from the electronic health record were tested for nonresponse bias and observable confounding. Following targeted learning-based nonresponse adjustment, procedure-specific opioid consumption quantiles (medians and 75th percentiles) were estimated and compared to a previous telephone-based reference survey.

**Results:**

6553 patients were included. Opioid consumption was measured in 44% of patients (2868), including 21% (1342) through survey response. Characteristics associated with inability to measure opioid consumption included age, tobacco use, and prescribed opioid dose. Among the 10 most common procedures, median consumption was only 36% of the median prescription size; 64% of prescribed opioids were not consumed. Among those procedures, nonresponse adjustment corrected the median opioid consumption by an average of 37% (IQR: 7, 65%) compared to unadjusted estimates, and corrected the 75th percentile by an average of 5% (IQR: 0, 12%). This brought median estimates for 5/10 procedures closer to telephone survey-based consumption estimates, and 75th percentile estimates for 2/10 procedures closer to telephone survey-based estimates.

**Conclusions:**

SMS-recruited online surveying can generate reliable opioid consumption estimates after nonresponse adjustment using patient factors recorded in the electronic health record, protecting patients from the risk of inaccurate prescription guidelines.

## Introduction

Overprescription of opioids after surgery poses a significant risk to patients and society [1]. In an effort to standardize prescribing practices and minimize overprescribing, many institutions have created guidelines based on expert clinical consensus to help surgeons determine the appropriate quantity of opioids to prescribe after a given procedure [2,3]. While an important first step, these consensus-based guidelines have been found to still result in significant excess opioid prescribing [2,3]. Recommendations regarding appropriate prescription quantities benefit from real-world evidence in the form of patient-reported, postdischarge opioid consumption data. Studies using opioid consumption data to guide post-surgical prescribing have shown significant improvement in opioid prescribing patterns [2,4–7]. However, only a few groups have collected this data at scale given the significant resources and labor required to collect patient-reported, postdischarge outcomes.

At our institution, we previously conducted a phone-based survey to collect post-discharge opioid consumption data from surgical patients. This involved research staff calling patients following discharge to inquire about opioid consumption [8]. This survey had an excellent response rate and the accuracy of such telephone-based post-surgical opioid consumption surveys has been validated elsewhere [8,9]. The consumption data obtained from phone surveys proved useful in guiding many institutional prescribing protocols and in evaluating the validity of existing guidelines and prescribing practices [2,8,10]. However, scaling the telephone-based method of data collection to additional procedures or institutions would have required a concomitant increase in staffing and related expenses. In an effort to reduce the manual component of data collection, we developed a new, automated method of data collection. We used a short messaging service (SMS)-to-web system, in which discharged patients were automatically sent a text message containing a link to a secure web survey, inviting patients to report their post-hospital opioid consumption on their phone. Prior studies have identified several perioperative patient- and procedure-specific factors associated with opioid consumption after surgery [11,12]. Some prior studies suggest that these factors are also associated with survey response [13–17]. Factors associated with both survey response and consumption (“confounders”) will distort survey results, potentially rendering estimates of typical opioid consumption less generalizable. Few prior opioid consumption surveys describe the characteristics of survey respondents in terms of these perioperative characteristics, or adjust for characteristics that may significantly alter survey results [11,12,18].

The objective of this study was to describe and evaluate the validity of our SMS-to-web-based system for collecting post-discharge opioid consumption data from surgical patients. In particular, we sought to accurately estimate typical consumption levels (medians and 75th percentiles) of opioids for common surgical procedures that could be used to guide future prescribing. We took a comprehensive approach to adjusting for nonresponse by describing the attributes of nonresponders using perioperative factors from the electronic health record (EHR) and then adjusting for those attributes to capture the complex relationship between health system data, survey response, and opioid consumption. The adjustment procedure relied on targeted learning, a machine learning-based causal inference methodology, which integrates semi-parametric efficiency theory with machine learning to reduce confounding bias and improve statistical power [19,20]. The adjusted estimates were compared to telephone-based consumption estimates, which serve as a reference standard (though with limitations, as we discuss). We hypothesized that adjusting for nonresponse – a step often overlooked in surgical survey research – would improve opioid consumption estimates by protecting against bias stemming from systematic variation in the types of patients who responded to the survey compared to those who did not.

## Methods

Following institutional review board (IRB) approval, we created a prospective, single-institution SMS-to-web-based survey system designed to measure post-discharge opioid consumption among surgical patients.

### Study cohort

Patients who underwent any surgical procedure at Beth Israel Deaconess Medical Center (BIDMC) from March 1, 2019 to October 31, 2020 were prospectively surveyed after discharge to assess the quantity of opioids consumed and to measure patient-reported satisfaction with pain control. Patients were included if they were 18 years old or greater, spoke English, had a valid cell phone number, and underwent any surgery at our institution during the study period. Patients were excluded if they were: trauma patients, patients undergoing multiple surgeries during their index hospitalization, hospitalized for greater than 2 weeks after their surgery, discharged to a rehab facility, were readmitted prior to completing the survey, incarcerated patients, or expired patients. Trauma patients and patients undergoing multiple surgical interventions were excluded given the multifactorial nature of pain and likely confounding of pain control requirements, due to multiple injuries, including non-operative injuries and operative injuries. The most frequently performed operations at our institution during the study period were: thoracic/lumbar/sacral discectomy, laminectomy and/or fusion; anterior cervical discectomy and fusion; thoracic/lumbar/sacral microdiscectomy; sternotomy; reduction mammoplasty; open inguinal hernia repair; cholecystectomy; laparoscopic appendectomy; carpal tunnel release; and thyroidectomy.

### SMS-to-web based survey and EHR-based data collection

**Fig. 1.**
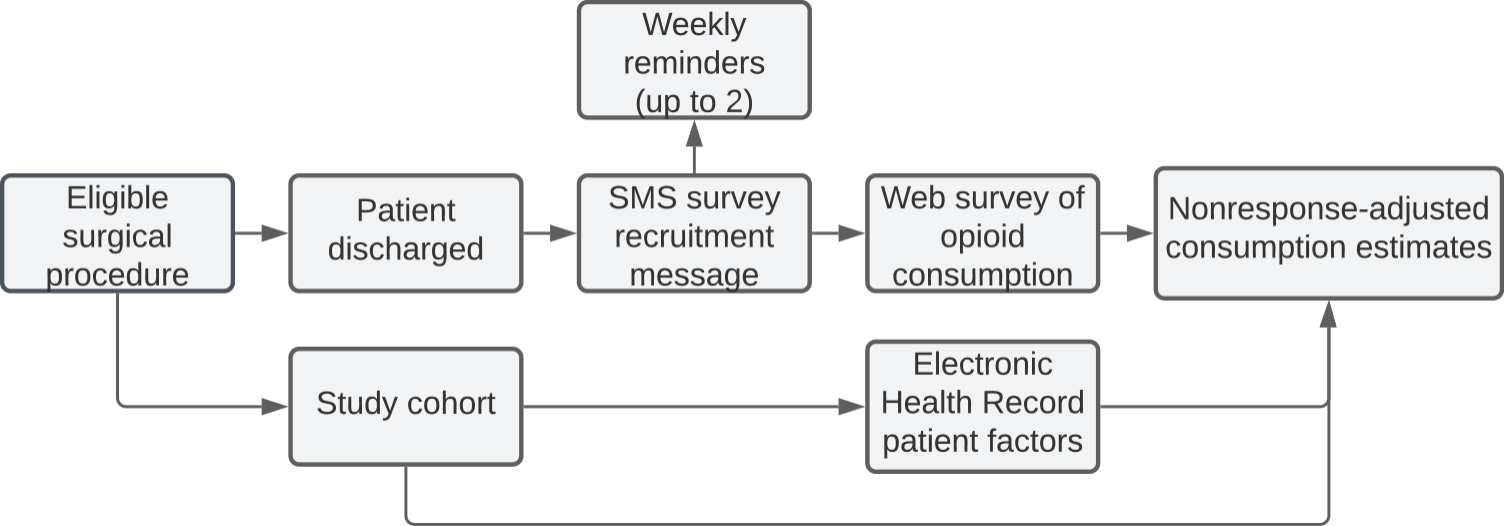
Workflow of the SMS-to-web patient outcome survey system.

Based on prior work by our group, we developed and implemented an automated, SMS-to-web based approach to collect patient reported opioid consumption. The novel survey instrument was informed by a prior validated phone survey and is provided in Supplementary Table S1. An initial SMS message was sent 14 days after discharge, and up to 2 reminder messages were sent to patients at 7-day intervals thereafter. Via the survey, patients were asked to report how many pills were remaining from their discharge prescription. For patients with no opioid prescription at discharge, as recorded in the EHR, the quantity of opioids consumed was designated as zero. The survey was hosted using Research Electronic Data Capture (REDcap) software [21]. The system design is summarized in Fig. 1.

### Study outcomes and covariates

The primary outcome was the number of opioid pills consumed by the patient after discharge from the hospital, converted to morphine milligram equivalents (MMEs). Initial opioid prescription size, patient demographics, comorbidities, hospitalization status, and surgical- and anesthesia-related characteristics were programmatically extracted from the EHR. Extracting EHR-based variables permitted nonresponse adjustment of our survey results so that we could more accurately estimate post-discharge opioid consumption. A summary of the 40 predictors is provided in Supplementary Table S2.

### Reference group: telephone-based estimates of opioid consumption

To provide a limited reference for comparison for our SMS-to-web survey, we used a prospective database of opioid consumption data derived from a previous telephone-based survey implemented at our institution from October 2017 to June 2018. Patients who underwent any surgery during that period were called 7 days after discharge from the hospital and queried about how many pills remained from their initial prescription. Patients who were still consuming opioids were called at 7-day intervals for up to 3 calls in order to record the final quantity of pills consumed. Of 3302 eligible patients, 1980 (60%) provided complete post-discharge opioid consumption information. Additional details, as well as the telephone-based survey instrument, are provided in a previous study [8].

### Statistical analysis

Variation in response by EHR characteristics was evaluated using chi-squared tests. Continuous variables were grouped into discrete bins based on common clinical thresholds (i.e.. BMI) or based on sample quantiles (i.e., age) so that different subgroups of these variables could be displayed; the Kolmogorov-Smirnov (K-S) test results on raw continuous variables were comparable. Missing predictor data was imputed using generalized low-rank models [22,23], and missingness indicators were included as predictors after eliminating perfectly collinear indicators [24]. Additional details on missing data imputation are included in the supplemental information (Supplementary Methods).

Nonresponse bias is a primary threat to survey-based data collection, where estimates of the outcome distribution (opioid consumption in our case) are confounded by variables that are related both to probability of responding to the survey and to the outcome [25,26]. We conducted a targeted learning (TL)-based double-robust analysis that reduces nonresponse-related confounding bias in three ways: (1) inverse probability weights based on a propensity score estimate, (2) an outcome regression adjustment that estimates the relationship between patient characteristics and opioid consumption, and (3) a targeting step that improves estimation using the equation of the specific statistical parameter being studied (i.e., quantiles in our case) [19,20]. We used this TL approach because quantile estimation tasks similar to the ones we performed in this study (i.e., providing an estimate of the median and 75th percentile of opioid consumption for guideline generation) based on targeted learning has shown efficiency gains (narrowing of confidence intervals) equivalent to doubling the number of survey responses [27].

Machine learning-based estimators have the potential to more accurately capture complex nonlinear relationships between predictor variables and survey response, thereby minimizing residual bias [28]. We used an ensemble machine learning approach called the Super Learner algorithm, with associated R package (version 2.0–28), to evaluate multiple prediction algorithms for their accuracy in capturing these relationships, and to create a weighted average designed to achieve the best accuracy [29–31]. The set of estimation algorithms, called the “library”, for the outcome regression (prediction of MMEs consumed, among those prescribed opioids) was: the outcome mean, stratification on prescribed MMEs and discharge day MMEs, ordinary least squares (OLS), lasso (glmnet package, version 4.1.2) [32], and random forest (ranger package, version 0.13.1) [33]. The latter three algorithms were tested with all predictors and with predictors restricted to those linearly correlated with the outcome at a *p*-value < 0.1. The SuperLearner library for the propensity score estimation (measurement of opioid consumption) was the same as the outcome regression, with the addition of Bayesian additive regression trees (BART) [34]. The latter five estimators (i.e. all except the outcome mean and stratified estimator) were tested with all predictors and with predictors restricted to those linearly correlated with the outcome at a *p*-value < 0.1. These modeled relationships were then used to generate quantile estimations using TL as described above.

Although targeted learning-based double robust adjustment can be performed solely with simpler logistic regression or linear probability models, their linear formulas oversimplify the complex relationship between predictor variables and our two outcomes of interest, opioid consumption and survey response [35,36]. Accepting this misspecification bias may distort consumption estimates further from the truth, leading to potential patient harm. If those linear algorithms are the best approximation to the data generating processes being modeled, ensemble estimators will automatically place high weight on their predictions. In other words, ensemble estimators that include standard linear algorithms will use those linear algorithms where they fit the data well, and will improve on them when they do not.

We evaluated the relative importance of predictors by tallying how frequently they were used in decision tree splits of the best performing machine learning algorithm, Bayesian additive regression trees (BART) [37]. All statistical analysis was performed using R (version 4.1.3). The study was reported in accordance with the Strengthening the Reporting of Observational Studies in Epidemiology (STROBE) guidelines.

## Results

**Fig. 2.**
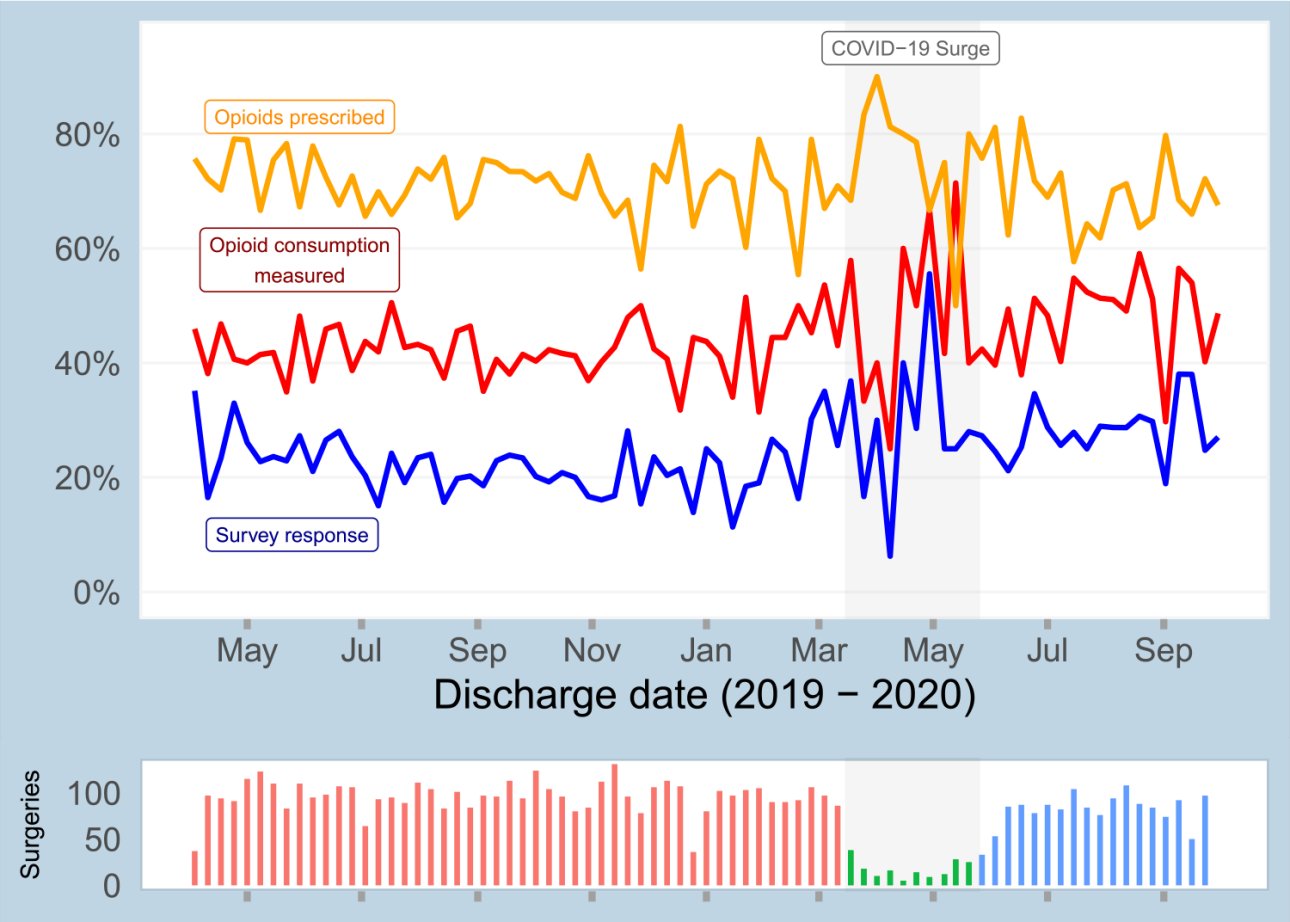
Temporal summary of study cohort, showing survey response rate, rate of being prescribed any opioids, and rate of opioid consumption being measured (i.e., non-missing).

6553 surgical patients were surveyed between March 1, 2019 to October 31, 2020 via the SMS-to-web based approach regarding their opioid consumption after discharge. Of these patients, 20.5% (1342) provided opioid consumption information in the web-based survey. By aggregating survey responses and data for patients with no opioid prescription, as documented in the EHR, we were able to measure post-discharge opioid consumption for 43.8% of participants (2868). A data flow diagram of the survey response analysis is shown in Supplementary Fig. S1. Baseline characteristics of included patients are shown in Table 1. Temporal trends in survey response rate, rate of being prescribed any opioids, and opioid consumption measurement rate are shown for the study cohort in Fig. 2.

**Fig. 3.**
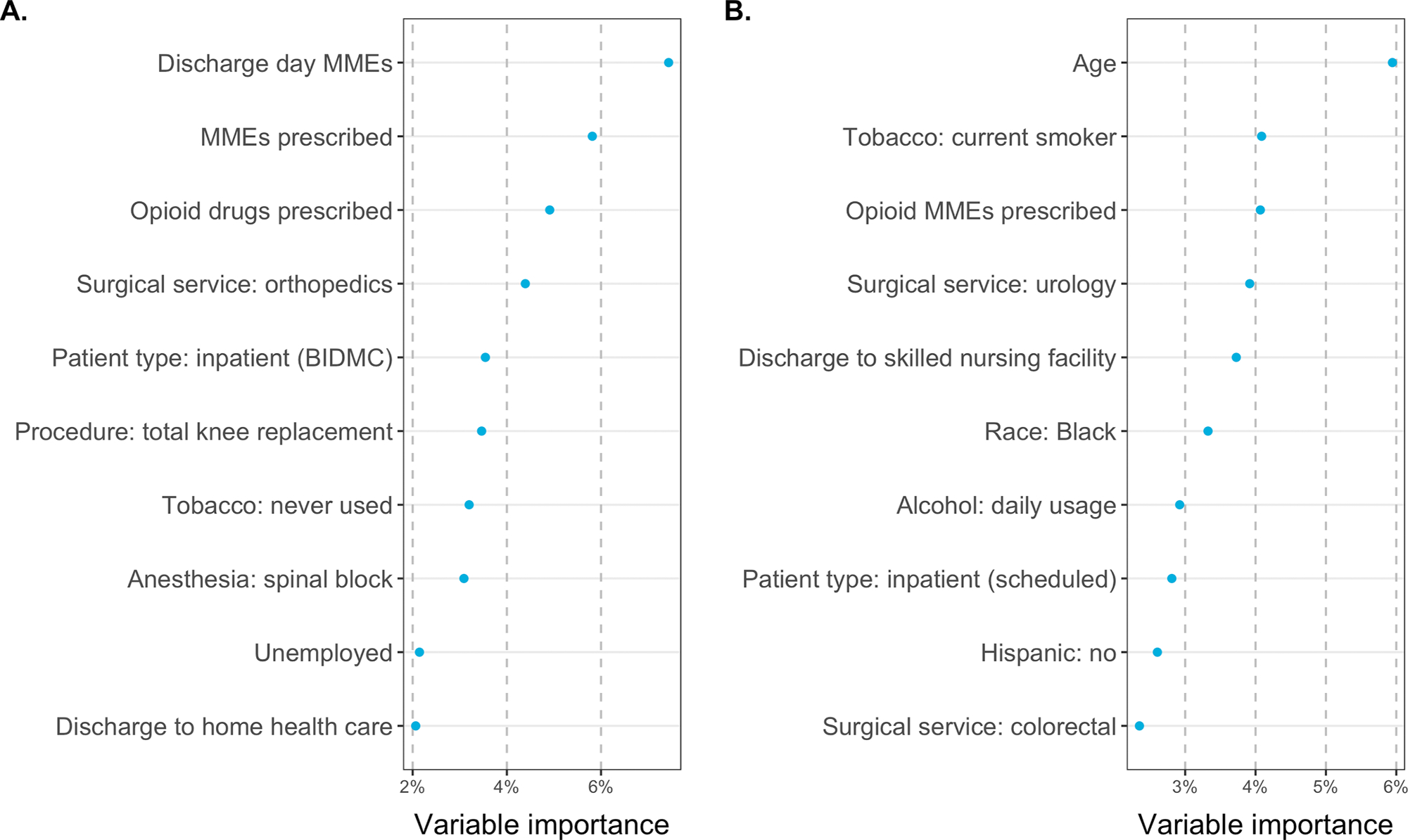
(a) Ranking of clinical and perioperative characteristics most associated with the ability to measure opioid consumption via survey or EHR based on BART, among patients prescribed opioids. (b) Ranking of EHR characteristics most associated with MMEs consumed among patients prescribed opioids. These include random forest-based feature screening to select the top 25 important variables, prior to indicator-encoding categorical variables.

The characteristics of survey responders and nonresponders and a bivariate analysis of select EHR-based clinical factors’ association with survey response are shown in Table 2. Characteristics significantly associated with survey response included age 61–70 years (24.7% response rate, *p* < 0.001), white race (22.6%, *p* < 0.001), length of stay 1–2 days (23.0%, *p* = 0.002), presence of a preoperative assessment visit (21.2%, *p* < 0.001), never smoker status (21.6%, *p* < 0.001), daily alcohol use (27.6%, *p* < 0.001), and American Society of Anesthesiologists (ASA) class 2 (22.5%, *p* = 0.001). Characteristics significantly associated with the ability to measure a patient’s opioid consumption (either through patient-report or through EHR data indicating no opioid prescription) included age 71–95 years (49.2% consumption measurement rate, *p* < 0.001), outpatient surgery (46.8%, *p* < 0.001), presence of a preoperative assessment visit (44.3%, *p* = 0.018), a history of tobacco use (46.2%, *p* < 0.001), and zero MMEs consumed in the hospital in the 24 h prior to discharge (i.e., zero discharge day MMEs) (52.4%, *p* < 0.001). Using BART analysis, the relative predictive importance of each clinical characteristic to consumption measurement is shown in Fig. 3b, revealing that age and smoking status are the two most important predictors of response among those analyzed.

Some of these predictors of response overlap with predictors of MME consumption, as identified by BART analysis (Fig. 3a), indicating likely nonresponse bias. This underscores the importance of performing nonresponse adjustment of the opioid consumption estimates, as discussed in detail in the discussion.

**Fig. 4.**
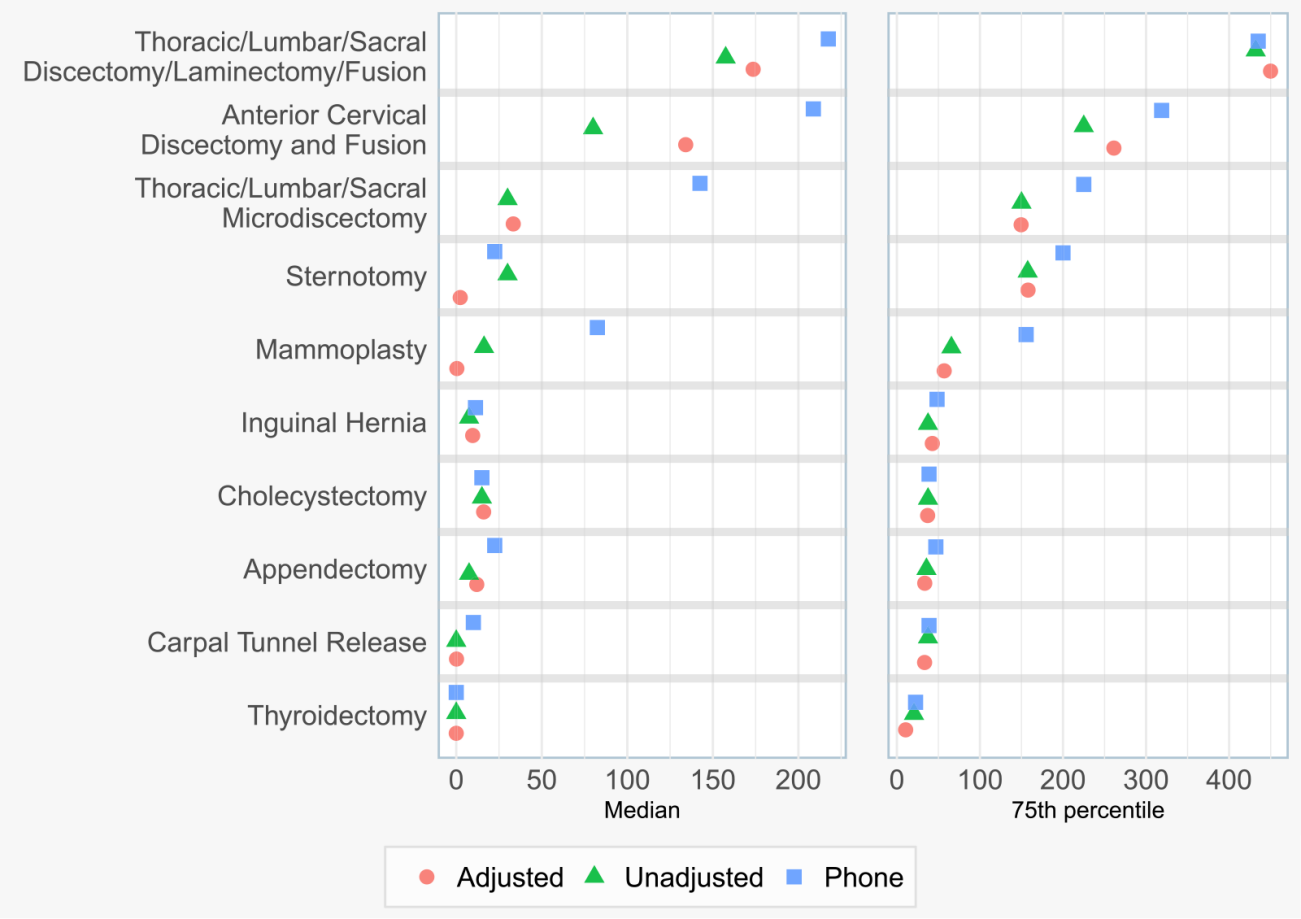
Opioid consumption in MMEs for the 10 most common surgical procedure groups by patient volume, examining the difference between nonresponse bias adjusted and unadjusted survey results, and comparing to the earlier phone survey.

Following TL-based nonresponse bias adjustment of our SMS-to-web data, we estimated the median and 75th percentile MME consumption for the 10 most common surgical procedures in our dataset, and compared them against telephone-based estimates (Fig. 4). TL nonresponse bias adjustment corrected median MME consumption estimates by an average of 37%, and corrected 75th percentile consumption estimates by an average of 5%, compared to the unadjusted survey results. This adjustment brought the median estimates for 5/10 procedures closer to telephone survey-based consumption estimates, and brought the 75th percentile estimates for 2/10 procedures closer to telephone survey-based consumption estimates.

Our findinges underscore the extent of overprescribing; across the top procedures, median consumption was only 36% of the median prescription size; 64% of prescribed opioids were not consumed. Additionally, 75th percentile consumption represented 72% of 75th percentile prescription size. A comparison of the demographics of SMS-to-web survey responders and telephone survey responders is shown in Supplementary Table S3. Procedure-specific point estimates of opioid consumption are provided in Supplementary Table S4.

**Fig. 5.**
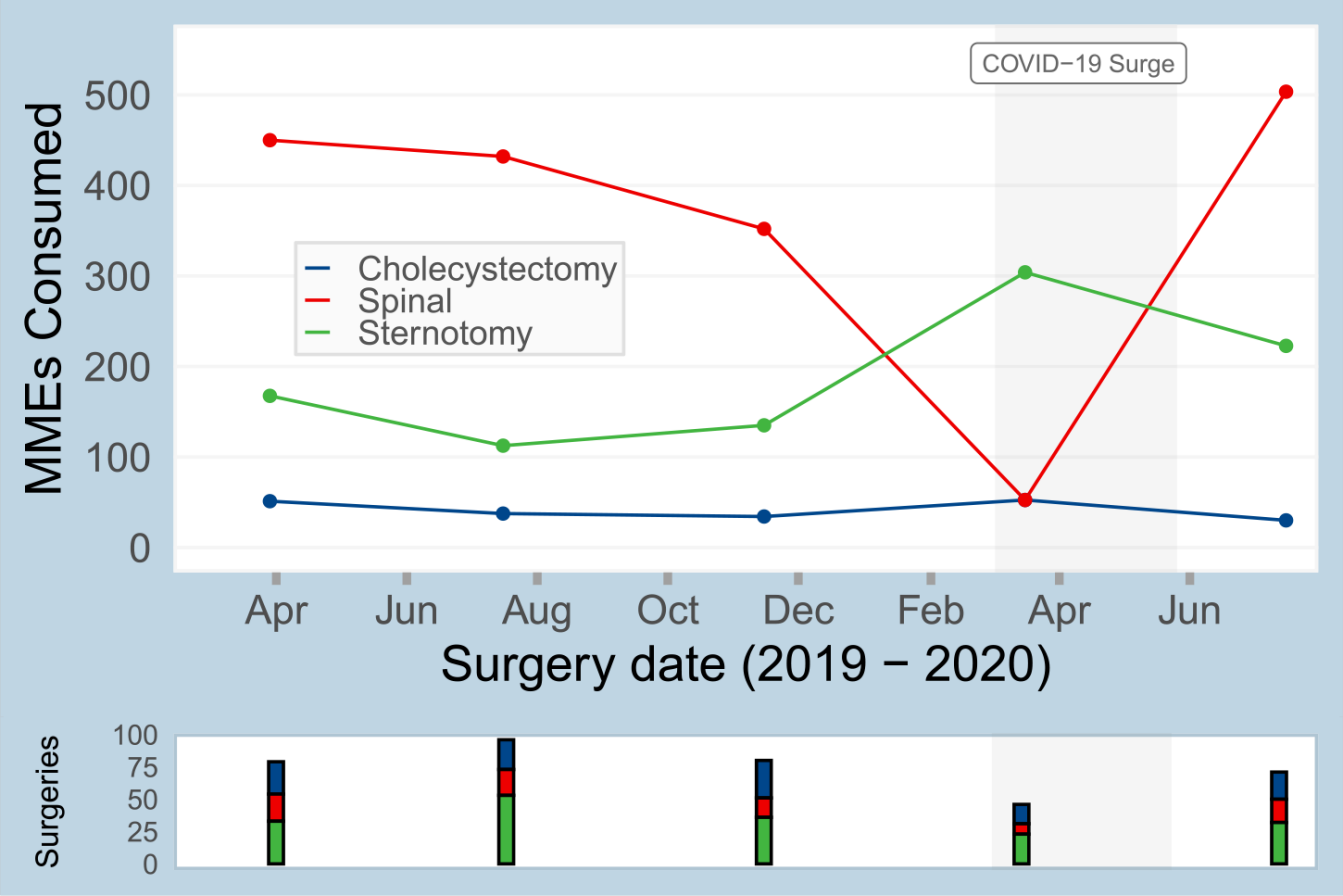
Temporal trends in the estimated 75th percentile of opioid consumption for 3 common surgical bins: sternotomy, cholecystectomy, and thoracic/lumbar/sacral discectomy/laminectomy/fusion (abbreviated as “spinal” in figure).

Changes in patient-reported opioid consumption over the course of the study period were identified. As shown for three representative surgeries in Fig. 5, the procedure-specific 75th percentile of consumption varied over the course of the study period. For example, the 75th percentile consumption for sternotomy was 168 MMEs in April 2019, 113 MMEs in July 2019, and 223 MMEs in July 2020. Given that these estimates of opioid consumption are used to inform opioid prescribing guidelines, evaluating temporal changes is critical, as we note in the discussion.

## Discussion

In this study, we examined the characteristics of an SMS-to-web survey to measure opioid consumption after discharge among post-surgical patients. We found that several clinical and perioperative factors were associated with both survey response and opioid consumption, suggesting that unadjusted survey measurements were biased (confounded). Using clinical factors found in the EHR to adjust for nonresponse protected against bias in opioid consumption estimates, and significantly changed typical opioid consumption estimates for more than half of the top 10 most frequently performed procedures. We observed steady decreases in opioid prescribing and patient’s post-surgical opioid consumption between the start of telephone-based data collection and the end of the study period in 2020, and thus, the observed differences in consumption measured via phone-survey and the SMS-to-web survey is likely exaggerated. However, nonresponse adjustment critically protects estimates of opioid consumption from probable nonresponse bias, and is an important step in producing reliable opioid consumption data to inform prescribing guidelines.

### Importance of nonresponse adjustment

Nonresponse bias is a factor in any survey-based data collection, and nonresponse adjustment has been a standard practice in survey research for decades [25]. Surprisingly, surgical studies often overlook the issue of nonresponse and do not correct for it [38,39]; failing to do this prior to interpreting survey results can lead to biased results, non-generalizable conclusions, and patient harm. To our knowledge, no opioid consumption surveys have adjusted their consumption estimates for nonresponse [11,12,18].

In the absence of adjustments for nonresponse, the accuracy of survey-based opioid consumption estimates is largely unknown; yet, this data is often the basis of clinical decisions. Few studies assessing opioid consumption have examined the factors associated with nonresponse. A recent study measuring post-discharge opioid consumption among patients undergoing orthopedic and urologic surgery using an SMS-based survey found that the presence of comorbidities were associated with nonresponse; however, nonresponse adjustment was not performed [18]. Other factors associated with survey response in regard to post-surgical opioid consumption are not known. The likely confounding of patient-reported opioid consumption by race deserves particular consideration; omitting nonresponse adjustment may contribute to known health inequities in pain treatment by race [40], along with reinforcing overarching issues of structural medical racism [41]. The importance of examining health equity in patient-reported outcomes has been noted in surgical research [42].

One complication of nonresponse adjustment is that for any given opioid consumption estimate for a surgical procedure, there may be little or no difference in the adjusted compared to the unadjusted results, which then begs the question of if it is a necessary step. But for a set of procedures (e.g. spinal surgery), we found large differences in the unadjusted and adjusted estimates. We consider this combination of scenarios to be the *paradox of nonresponse adjustment benefit*: for many estimates there may be no clinically relevant benefit to nonresponse adjustment, while for others they may be substantial improvements in accuracy, but the only way to find out if nonresponse adjustment is important for a given consumption estimate is to conduct the adjustment for all procedures.

### EHR-based nonresponse adjustments

We found that several EHR-based clinical and perioperative factors were associated with both opioid consumption and survey response in our study, suggesting that unadjusted estimates of opioid consumption may be biased due to confounding. Multiple prior studies confirm our findings that age, race, smoking status, and comorbidity burden are associated with MME consumption [11,12,43]. While prior studies on non-opioid related topics have uncovered associations between EHR-based clinical factors and survey response [13–17], the association of these clinical and perioperative attributes with both survey response and post-surgical opioid consumption is novel and indicative of observed confounding, which may lead to inaccurate consumption estimates if not incorporated into the statistical adjustment procedure. Failing to perform nonresponse adjustment, or adjusting only with demographic factors, may adversely affect patient care if the estimates are used to guide opioid prescribing.

For the most common procedures at our institution, nonresponse adjustment using relevant EHR-derived attributes revised the SMS-to-web survey derived median and 75th percentile opioid consumption estimates, often in the direction of previously validated estimates from phone surveys. Previous studies have shown that the median opioid consumption for many common surgical procedures is relatively small (e.g. three 5 mg oxycodone tablets) and thus, minor corrections in opioid consumption estimates, secondary to nonresponse adjustment, are clinically relevant [2]. While the adjustments and comparisons to the phone survey estimates must be considered in the context of broader consumption and prescribing trends, as highlighted in Fig. 5, nonresponse adjustment protects against potential errors related to response bias. Assessing for nonresponse bias is a critical step in clinical research related to patient-reported survey data; particularly when nonresponse bias is observed, nonresponse adjustments should be completed to improve study accuracy.

In addition, we used EHR data to improve the completeness of our consumption data, rather than relying on survey-reported opioid consumption alone. Patients who did not respond to the survey but were not prescribed any opioids at discharge, as documented in the EHR, were counted as consuming zero MMEs in our final dataset. In integrating survey data with EHR-derived data, we were able to increase the number of patients for whom opioid consumption could be measured. Given the realities of post-surgical opioid consumption, in which a large proportion of patients do not consume any opioids after discharge, it is highly valuable that the opioid consumption of this subset of patients can reliably be abstracted from the record. By its nature, inclusion of the EHR-derived opioid consumption data does not mitigate the need for nonresponse adjustment.

### Importance of machine learning-based adjustment

Traditional (parametric) methods for nonresponse bias adjustment such as logistic regression are limited in their ability to correct for bias because they only partially capture the complex patterns that differentiate responders and nonresponders [35,36]. The residual bias derived from these simple models may distort opioid consumption estimates from the true underlying patient consumption, leading to patient harm by misinforming prescribing guidelines. Ensemble machine learning, as we used in this study, can reduce estimation bias compared to logistic regression by relying on algorithms that best predict the outcome and response variables [28,44]. In observational studies at risk of confounding bias, it is a best practice to use machine learning-based adjustment to generate the least biased estimates. Future clinical research using patient-reported outcomes and/or survey data should incorporate these recent methodological developments [45]. However, while the techniques employed in the current study are generally superior to parametric methods (i.e. have greater asymptotic efficiency), correction for nonresponse using parametric methods may achieve a substantial portion of the potential benefit from more complex approaches.

### Limitations

While our prior telephone-based survey results served as a limited standard of comparison for our SMS-to-web survey results, there is no “gold standard” survey method for measuring opioid consumption. Telephone-based consumption estimates remain vulnerable to nonresponse bias, and in our case the temporal difference in the two datasets means that the telephone-based estimates are influenced by historically higher opioid prescribing. In the absence of a gold standard, no study can prove the benefit of performing nonresponse adjustment; however, nonresponse adjustment of survey responses is inherently beneficial to account for systematic differences in response attributes. In addition, we identified several clinical and perioperative factors that were associated with both survey response and opioid consumption, indicating the probable benefit of nonresponse adjustment due to confounding.

Furthermore, there were background trends in opioid prescribing and opioid consumption at our institution between 2017–2018 when the telephone data was collected, and 2019–2020 when the SMS-to-web data was collected. The factors underlying this trend reflect patient-level changes, societal-level changes, short- and long-term effects of the COVID-19 pandemic, and broader efforts at our institution to improve postsurgical opioid use. As our study and prior work has identified, one of the strongest predictors of opioid consumption is prescription size [11]. The difference between SMS-to-web consumption estimates and telephone consumption estimates, even after nonresponse adjustment, may be partially explained by these two phenomena. As opioid consumption estimates are used to inform opioid prescribing guidelines, this analysis highlights the importance of updating metrics in response to observed behavior changes related to postsurgical opioid consumption. Nonetheless, we chose to use our telephone-based consumption data as a reference for comparison since it is a commonly used method for collecting opioid consumption data in prior studies, had a high response rate of 60% in our study, and its accuracy has been validated by previous work demonstrating that it aligns closely with in-person based consumption estimates [8,9,11,12]. While our SMS-based survey had a comparatively lower response rate as expected [18,46,47], the automated nature of this survey method may limit recall bias. We were able to automatically contact patients on a weekly basis after discharge, while studies using telephone and other labor-intensive methods have waited up to 12 months after discharge to survey patients on pain control and opioid consumption [38].

We also acknowledge that our study excluded patients who did not speak English or did not have access to a cellular phone. While additional techniques can be used in the future to survey this subset of patients, this limitation highlights the importance of conducting nonresponse adjustment of our SMS-to-Web-based opioid consumption estimates. In addition, trauma patients and patients who underwent multiple procedures were excluded given the multifactorial nature of their pain and inability to associate their opioid consumption with a single intervention or injury. Furthermore, given statistical constraints, our study only examined the role of performing nonresponse adjustment for top 10 most common procedures at our institution; additional research is needed to describe and evaluate the nonresponse adjustment in other procedures. A general limitation of procedure-agnostic surgical research, such as this study, is the wide range of distinct surgical procedure bins, even after grouping similar procedures, which constrains statistical power for procedure-specific opioid consumption estimates and may limit the impact of nonresponse adjustment compared to larger sample sizes. Multi-institutional data sharing may be necessary to fully benefit from procedure-specific nonresponse adjustment for patient-reported outcomes. Lastly, while not an aim of our current study, further research is needed to understand how changes in opioid prescribing guidelines and opioid consumption relate to patient-reported pain control.

## Conclusion

In this work we have described an automated method for collecting post-discharge opioid consumption data from surgical patients using an SMS-to-web survey system, followed by nonresponse adjustment using EHR-sourced factors when estimating procedure-specific consumption quantiles. Patient-reported outcomes, such as opioid consumption, are vulnerable to confounding bias due to patterns in nonresponse, but the wide variety of clinical factors available in the EHR makes these patterns observable and correctable using statistical adjustment. The ease of data collection using a nonresponse-adjusted SMS-to-web survey may enable large-scale opioid consumption data to guide future opioid prescribing guidelines, allowing postoperative care to move beyond less effective consensus guidelines that risk perpetuating overprescribing. While opioid consumption estimates for any particular procedure may change little between unadjusted and adjusted versions, our finding that certain procedures had large corrections from nonresponse adjustment imply that such adjustment should be routinely employed as a prophylactic measure.

## Supplementary Material

2

1

## Figures and Tables

**Table 1:** Baseline characteristics of study participants: all study participants, survey responders, and those for whom opioid consumption could be measured either through survey response or EHR data.

Characteristic	Value	Full study (*n* = 6553)	Survey responders (*n* = 1342)	Opioid consumption measured (*n* = 2868)

Age	18 – 45	24.0%	19.0%	21.9%
	46 – 60	31.9%	33.8%	30.9%
	61 – 70	26.1%	31.4%	27.0%
	71 – 95	18.0%	15.8%	20.3%
Missing preoperative assessment	No	86.5%	89.8%	87.7%
	Yes	13.5%	10.2%	12.3%
Race	White	72.9%	80.6%	74.0%
	Asian	2.6%	2.4%	2.8%
	Black	11.1%	6.7%	9.9%
	Hispanic	1.9%	1.1%	2.0%
	Other	11.5%	9.2%	11.3%
Length of stay	Outpatient	41.7%	39.9%	44.6%
	1 – 2 days	31.1%	35.0%	30.9%
	3 – 15 days	27.1%	25.1%	24.5%
Tobacco use	Never used	54.9%	55.9%	55.2%
	Within past month	9.2%	5.9%	7.4%
	Within 1–12 months	3.0%	2.8%	3.1%
	History	32.9%	35.4%	34.3%
Surgical service	Other	57.4%	62.4%	58.4%
	Orthopedic	30.3%	28.9%	28.4%
	Colorectal	4.8%	3.3%	3.6%
	Transplant	4.3%	3.4%	4.7%
	Vascular	3.2%	2.0%	4.9%
Opioid drugs prescribed	0	29.0%	28.2%	66.3%
	1	68.8%	70.1%	32.9%
	2 or 3	2.2%	1.6%	0.8%
Opioid MMEs prescribed	0	29.1%	28.3%	66.3%
	1 – 99	30.6%	31.5%	14.8%
	100 – 299	24.1%	25.1%	11.7%
	300+	16.3%	15.2%	7.2%

**Table 2:** Bivariate analysis of clinical and perioperative factors associated with ability to measure opioid consumption, either through survey response or the combination of survey response and EHR data (measurement).

Characteristic	Value	N	Response %	Response *p*-value	Consumption measured %	Consumption *p*-value

Age, years	18 – 45	1,573	16.2%	<0.001	39.9%	<0.001
	46 – 60	2,089	21.7%		42.4%	
	61 – 70	1,709	24.7%		45.3%	
	71 – 95	1,182	17.9%		49.2%	
Sex	Female	3,422	21.2%	0.146	44.9%	0.059
	Male	3,131	19.7%		42.5%	
Race	White	4,774	22.6%	<0.001	44.4%	0.086
	Asian	170	18.8%		47.1%	
	Black	727	12.4%		39.2%	
	Hispanic	127	11.8%		45.7%	
	Other	755	16.4%		42.9%	
Length of stay	Outpatient	2,735	19.6%	0.002	46.8%	<0.001
	1 – 2 days	2,041	23.0%		43.4%	
	3 – 15 days	1,777	19.0%		39.6%	
Preoperative assessment	Observed	5,671	21.2%	<0.001	44.3%	0.018
	Missing	882	15.5%		40.0%	
Tobacco use	Never used	3,111	21.6%	<0.001	44.6%	<0.001
	Within past month	522	13.6%		35.6%	
	Within 1–12 months	171	19.9%		45.0%	
	History	1,867	22.9%		46.2%	
Opioid MMEs prescribed*	0	1,901	19.9%	0.452	100.0%	0.440
	1 – 99	2,001	21.1%		21.2%	
	100 – 299	1,576	21.3%		21.3%	
	300+	1,065	19.2%		19.4%	
Alcohol use	Never	1,611	17.9%	<0.001	42.8%	0.198
	Occasionally	3,582	21.9%		44.7%	
	Daily	478	27.6%		47.1%	
Discharge day MMEs	0	4,196	20.3%	0.275	52.4%	<0.001
	1 – 10	1,832	21.5%		29.3%	
	> 10	525	18.5%		25.7%	
ASA class	1	526	17.9%	0.001	41.4%	0.330
	2	3220	22.5%		43.3%	
	3	2288	18.6%		45.1%	
	4–5	512	18.9%		42.8%	

* The consumption chi-squared test and *p*-value excludes the patients with no opioids prescribed, as they have 100% consumption measurement rate.
